# Visually Assessing Equine Quality of Movement: A Survey to Identify Key Movements and Patient-Specific Measures

**DOI:** 10.3390/ani13182822

**Published:** 2023-09-05

**Authors:** Annette G. Bowen, Gillian Tabor, Raphael Labens, Hayley Randle

**Affiliations:** 1 School of Agricultural, Environmental and Veterinary Sciences, Faculty of Science and Health, Charles Sturt University, Wagga Wagga, NSW 2678, Australia; rlabens@csu.edu.au (R.L.); hrandle@csu.edu.au (H.R.); 2 Equestrian Performance Research Centre, Hartpury University, Gloucester GL19 3BE, Gloucestershire, UK; gillian.tabor@hartpury.ac.uk

**Keywords:** equine physiotherapy, quality of movement, outcome measure, rehabilitation, goal setting

## Abstract

**Simple Summary:**

Physiotherapy and rehabilitation is a burgeoning area of practice; to be evidence-based, it needs outcome measures designed for its focus on function. Based on frequency of use and rationale, this online survey aimed to identify a core group of in-hand assessments for equine movement. Additionally, the survey gathered information on how movement is currently monitored and opinions on the usefulness of modifying a patient-reported outcome measure for equine use. The survey attracted 81 participants and identified 24 key movements, including walk and trot on both firm and soft surfaces in a straight line and on a small circle, plus step back, hind leg cross-over, transitions and lunging at walk, trot and canter. Access to suitable surfaces and the training level of the horse and handler are the main barriers to using other movements. The majority (82%) of survey participants agreed or strongly agreed that a modified Patient-Specific Functional Scale would be useful for measuring complex movements. This knowledge of how equine clinicians are currently monitoring movement and using goal-setting will assist in designing a new outcome measure for quality of movement that includes both standardised and individualised measures.

**Abstract:**

Outcome measures are essential for monitoring treatment efficacy. The lack of measures for quality of movement in equine physiotherapy and rehabilitation impairs evidence-based practice. To develop a new field-based outcome measure, it is necessary to determine movements most frequently observed during assessment of rehabilitation and performance management cases. An online survey of 81 equine sports medicine veterinarians and equine allied-health clinicians was conducted. The key movements identified included walk and trot on both firm and soft surfaces in a straight line and on a small circle, plus step back, hind leg cross-over, transitions and lunging at walk, trot and canter. The main barriers to observing some movements are access to suitable surfaces and the training level of the horse and handler. Subjective visual assessment of live or videoed horses was the most common method used to track progress of complex movements. The majority (82%) of survey participants agreed or strongly agreed that a modified Patient-Specific Functional Scale would be useful for measuring complex movements. Comments from all professions show a desire to have outcome measures relevant to their needs. This survey identified 24 in-hand movements, which can be used to form the foundation of a simple field-based outcome measure for quality of movement.

## 1. Introduction

Movement dysfunction causes horses significant suffering, therefore managing it efficiently is critically important. Because the body prioritises function over pain [1], addressing movement dysfunction is essential for positive animal welfare [2]. Movement dysfunction refers to poor quality of movement, and considers aspects such as whole body biomechanics, gait pattern, symmetry, motor-control, muscle activity and timing, willingness, and behaviours indicating discomfort [3,4]. Since physiotherapy is centred around improving function [5,6], physiotherapists are ideally placed to treat horses presenting with movement dysfunction.

Outcome measures that reflect client health status are essential for monitoring the safety and efficacy of treatment [7,8]. The absence of even a simple tool to quantify the functional change in response to treatment has allowed the use of non-evidence-based therapies to proliferate within the equine industry [9]. Ineffective treatments likely have negative welfare implications, wasting owners’ time and money, while prolonging equine pain and dysfunction. Valid, reliable and relevant outcome measures that can be used in the field to assess quality of movement in horses and to monitor the success of equine physiotherapy and rehabilitation are needed [7,10].

Having been designed to grade lameness when walking and trotting in a straight line, existing lameness scales are too broad to adequately document subtle changes in quality of movement [3,11,12]. Given that these lameness scales are also subject to inconsistent applications by different practitioners [13,14,15,16], the lack of agreement between veterinary experts on the definition and measurement of lameness and asymmetry is not surprising [17]. Trot in a straight line, on its own, is insufficient to assess quality of movement due to the complexity and task-specific nature of movement [18]. Differences have been identified in hoof loading, spinal kinematics, body lean and asymmetry between different gaits, figures and surfaces [19,20,21,22,23,24,25,26,27]. Many movements tests are taught as an iterative assessment process [28]; however, it is currently unknown which ones are used most frequently in clinical practice, and if there is an established core group.

Instrumented gait analysis relying on inertia measurement units (IMUs) as used in the Equigait^®^ (Cheshunt, UK) or Equinosis Q^®^ (Columbia, IN, USA) system can objectively identify minute movement asymmetries. Despite acknowledging the value of IMUs, their data should be used as an aid to diagnosis [3,29], similar to medical imaging, and the clinical significance should be interpreted in conjunction with other assessments. While research using IMU’s is rapidly advancing, in the authors’ experience in the field, visual observation of movement still predominates, and this is routinely scored with subjective lameness grading scales.

While veterinarians need lameness scales to direct the clinical pathway for diagnosis, typically identifying a limb, severity and a pathoanatomical source [4,5], physiotherapists tend to use lameness scales as a triage tool. In addition to lameness scales there is also a place for a quality of movement outcome measure. Given that most horses are referred for physiotherapy with an established pathoanatomical diagnosis, the physiotherapists carry out a functional assessment to identify target tissues and directions for desensitising or strengthening (e.g., a cervical vertebral joint stiff into left lateral flexion, with hypertonicity of the right neck muscles and weak left neck muscles) [5].

Physiotherapists have a duty of care to ensure they provide quality practice; however, without outcome measures, decision-making is subject to bias and guesswork [30,31]. There is a strong desire from all stakeholders to have relevant outcome measures and research on the efficacy of equine physiotherapy [8,32,33]. However, current outcome measures designed for veterinarians that focus on lameness do not meet the needs of physiotherapists and equine clinicians working with rehabilitation and/or poor performance cases. Impairments are problems in body structure or function, while activity limitations refer to higher-level functions, such as complex whole-body movements [34]. Measures for both impairments and activity limitations are needed to accurately describe patient status [35]. While there are a variety of suitable equine impairment measures such as goniometry [36], palpation [37] and back profiles [38], there is currently a lack of outcome measures capable of monitoring activity limitations in horses. Without these, equine clinicians are left relying on subjective visual assessment and measures of impairment (e.g., passive range of movement), which should not be generalised to claim changes in whole-body movement.

For complex whole-body movements, goal setting is sometimes used in place of precise measures. In human physiotherapy, goal setting with the client is a vital part of client-centred care, and is used to monitor the long-term impact or value of care [39]. The Patient-Specific Functional Scale (PSFS) [40] (see Appendix A) is a patient-reported outcome measure used in human physiotherapy, which includes the client in scoring their ability to perform personally meaningful tasks. Part of the PSFS’s popularity may be due to its adaptability for a large variety of movements or goals, compared to exhaustive list-style patient- or owner-reported outcome measures, which are often time-consuming and contain many irrelevant questions [41]. It should be possible to modify the PSFS for use by owners/riders and clinicians in equine physiotherapy and rehabilitation. Past publications shed little light on methods used in equine practice to measure complex movements; therefore, it is necessary to investigate how equine clinicians are currently using goals and monitoring complex movements.

To address these problems, we propose a new outcome measure be developed for equine quality of movement. The Equine Musculoskeletal Rehabilitation Outcome Score (TEMROS) identified domains for a composite rehabilitation outcome measure, noting outcome measures are lacking for the performance/functional capacity domain and suggesting developing a battery of movement tests [42]. The proposed outcome measure would be for use by equine clinicians working with horses undergoing rehabilitation or managing performance issues. Such horses may present with movement dysfunction, motion asymmetry, inconsistent, or subtle or mild lameness (e.g., AAEP < 2: Lameness that is difficult to observe at a walk or trot in a straight line but is present under certain circumstances [43]). It is currently unknown which movements clinicians believe to be most important or useful to include when assessing quality of movement in equine rehabilitation and performance. The proposed outcome measure would be a combination of a standardised battery of key movement tests (for simple routine movements) supplemented with a client-specific measure (for bespoke complex movements) that can be integrated into the assessment process.

This survey was an early investigative step in the development of a new field-based outcome measure. The primary aim of the survey was to identify which in-hand movements equine clinicians observe most frequently, and if there is a key group that could be taken forward to develop a new quality of movement outcome measure for use in equine physiotherapy and rehabilitation. Additionally, this study aimed to gather information on how complex movement is currently monitored and opinions on the usefulness of modifying the PSFS.

## 2. Materials and Methods

### 2.1. Subjects

For this study, equine clinicians were defined as equine sports medicine and rehabilitation veterinarians and qualified equine allied health professionals. Qualifications/syllabus of equine clinicians are heterogenous worldwide, and so it logically follows this would affect clinical practices. Therefore, it was decided to set tight inclusion criteria to try and achieve more homogeneity in qualification types and practice. The following inclusion criteria were applied: equine clinicians experienced in the areas of rehabilitation and performance, with at least an undergraduate degree in a relevant field (qualification names vary worldwide). Post-graduate training was desirable to try to achieve similarity in clinical reasoning and assessment processes due to comparable academic backgrounds.

There is an unknown number of equine clinicians with post-graduate qualifications worldwide. While it is challenging to ascertain the population, response rate and study power for web-based surveys, a detailed web-based investigation was conducted to attempt to estimate the sample frame; this suggested 21 professional associations could provide coverage of approximately 1000 equine clinicians. The *a priori* calculation of study power (95% CI, 5% error) yielded a requirement of 278 responses. However, based on the generally very low response to online surveys [44] and a previous online survey of a subgroup of this population gathering only 71 responses [32], a 90% CI and 10% margin of error were applied for this study, reducing the required minimum sample size to 64. It must be highlighted that this calculation is only an estimate—the results are largely descriptive and should be interpreted in the context of the participants, and not generalized too broadly. Additionally, the authors adhered to the Checklist for Reporting Results of Internet E-Surveys (CHERRIES) where possible [44].

Participant recruitment was via equine clinicians’ professional associations (n = 21) who distributed the survey link through their member networks, e.g., Equine Sports Medicine and Rehabilitation Diplomats, Animal/Veterinary/Equine Physiotherapists and Animal Biomechanical Professionals (Chiropractors, Osteopaths), and flyers were posted on the associations’ public Facebook^®^ groups. Demographic information was collected on participant country of practice, qualifications, years of experience, current case load, and use of instrumented gait analysis. These details were used to determine eligibility for participation and to analyse variability in the sample’s responses based on participant’s background.

### 2.2. Data Collection

The questionnaire was conducted online using the Survey Monkey ^®^ platform. Questions were reviewed by Charles Sturt University’s (CSU) Spatial Data Analysis Network (SPAN), before being pilot-tested for user-friendliness by a small convenience sample of invited participants (n = 10). Pilot data were not included in the study results. Further revisions were made and the survey received ethical approval from Charles Sturt University, Human Research Ethics Committee (Protocol #H22082).

The questionnaire comprised 21 questions (7 open text, 2 closed-binary, 7 closed-Likert scale and 5 semi-closed) in three sections: 1—demographic details, 2—the frequency of movements used, and 3—how functional movement is currently monitored, with questions about goal setting, owner reported measures and the patient-specific-functional-scale. For Section 2, a list of 38 movements used during clinical assessment was compiled from published literature by the primary researcher (AB). To indicate how frequently each movement was used, participants had four possible responses: “always”, “often”, “sometimes” or “never”. The PSFS was included as a display item alongside the questions relating to it. Open text questions allowed participants to provide a rationale for their answers. The full questionnaire and answer options are available in the Supplementary Materials.

The survey was designed to be completed in 15 to 20 min and was open for 6 weeks from 23 May to 4 July 2022 inclusive. Reminders were emailed regularly to the associations and posted on their social media pages to encourage participation by their members.

The survey’s online landing page provided participant information and collected informed consent. Participation in the survey was voluntary and no incentives were offered. Anonymized survey data were received by the primary researcher (AB) and those requesting a summary of results were asked to directly email AB. The survey data were stored in accordance with the approved Human Research Ethics Application data management plan.

### 2.3. Data Analysis

Unusual responses were investigated before being included or excluded from the data, and the screened data were then collated by question in Microsoft Excel prior to being subjected to descriptive analysis. Jeffrey’s Amazing Statistics Program (JASP version 16) was used for inferential analysis.

For descriptive analysis, mean and standard deviation (SD) were calculated for years of practice. Percentages were calculated for undergraduate and post-graduate qualifications, country of practice, workload with humans, equines and canines/other species, frequency of seeing performance management and rehabilitation cases, frequency of using simple video, kinematics, IMU or other devices, movements used “always” or “often” combined, use of formal goal setting, use of owner-reported measures, familiarity with and use of the PSFS, opinion on usefulness of a modified PSFS for use by clinicians, owners (ADLs) and owners (complex movements), and participants based on background training. Mode was determined for the ordinal data relating to frequency of use of each movement.

For inferential analysis, Chi-squared tests were used to test for relationships between clinician background and the following: frequency of seeing rehabilitation cases, frequency of seeing performance cases, frequency of using simple video/kinematics/IMU/other devices, frequency of use of movement tests (then post hoc z scores manually calculated), use and familiarity with PSFS and opinion on usefulness of modified PSFS. Due to concerns on the violation of the normality assumption condition, the non-parametric Kruskal–Wallis test was used to test for relationships between clinician background and years of experience and proportion of work with human/equine/canine, plus between years’ experience and familiarity with PSFS. Statistical significance was determined by *p* < 0.05. NVIVO was used to assist the qualitative analysis of themes from the open-ended questions.

## 3. Results

### 3.1. Responses

The questionnaire attracted 90 respondents; however, 9 were excluded for not meeting the inclusion qualifications and/or incomplete responses (completion rate 94%). Responses from 81 participants were therefore included in the analysis, of which 73 completed all questions (completeness rate of 90%). Not all participants responded to every question; the following results are presented based on the number of responses for each question. While it is impossible to calculate response rate when distributing surveys online, of the 52 addresses emailed, 50 were received. Several associations replied that they were unable to distribute surveys to their members due to internal policies, or requested payment. In response to this, the sample frame was revised down to 700 equine clinicians, the resultant sample size (90% CI, 10% error) being 62 participants. Reminders were emailed to active email addresses representing 19 associations and regularly posted on association Facebook pages (n = 14). Based on the estimated sample frame the approximate response rate was 13%.

### 3.2. Subjects’ Background

Participants reported undergraduate qualifications in human physiotherapy (59%, n = 48), veterinary medicine (27%, n = 22), veterinary physiotherapy (10%, n = 8), human chiropractic (5%, n = 4), human osteopathy (5%, n = 4), and other (7%, n = 6) (zoology, biology, equine science, physical education, agriculture and manual therapy). Post-graduate qualifications included masters in veterinary/animal/equine physiotherapy (27%, n = 22), post-graduate diploma in veterinary/animal/equine physiotherapy (22%, n = 18), certificate or diploma in equine physiotherapy or rehabilitation (19%, n = 15), masters or post-graduate diploma in veterinary/animal/equine chiropractic (12%, n = 10), veterinary sports medicine and rehabilitation diploma (11%, n = 9), post-graduate diploma in animal biomechanical area (10%, n = 8), certificate or diploma in veterinary/animal/equine osteopathy (6%, n = 5), and other (14%, n = 11) (PhD, equine orthopaedics, equine surgery, veterinary acupuncture, equine biomechanics and rehabilitation, equine craniosacral therapy, equine anatomy and physiology, doctor of veterinary medicine, equine science).

Participants commonly held multiple qualifications, with 12% (n = 10) indicating more than one undergraduate qualification and 20% (n = 16) reporting more than one post-graduate qualification. Only 2% (n = 2) reported no post-graduate qualification.

Participants practiced as equine clinicians for a mean of 13.9 years (SD = 9.3). The majority of participants practiced in Australia (35%, n = 28), with 16% (n = 13) from the United Kingdom, 14% (n = 11) from the United States of America, 10% (n = 8) from New Zealand, 11% (n = 9) from mainland western European countries (Finland, Belgium, France, Germany, Italy, Netherlands and Sweden) and 13% (n = 10) from other countries (Ireland, Canada and South Africa).

**Figure 1:**
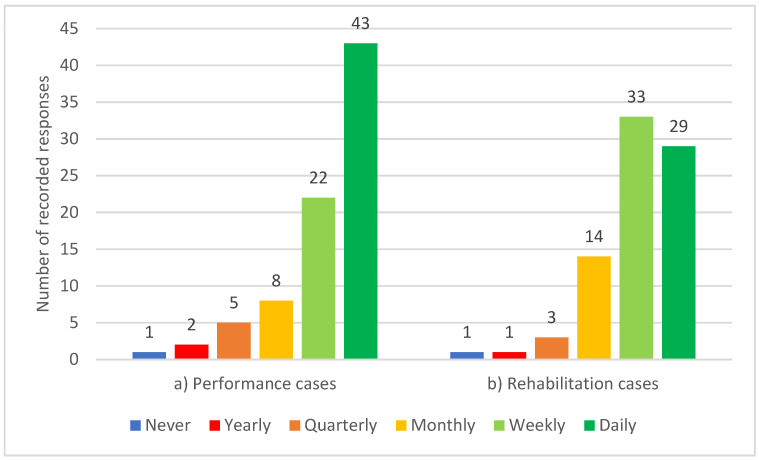
Frequency of participating equine clinicians seeing horses for (**a**) performance management and (**b**) rehabilitation (n = 81).

All participants reported working with horses, with almost one-third, 29% (n = 17), focusing solely on horses, 57% (n = 46) also working with dogs or other species, and 64% (n = 52) having performed some work with human clients. As a proportion of their workload, horses ranged from 1 to 100%, humans from 0 to 99% and dogs or other species from 0 to 70%. For all participants, the mean proportion of work with equids was 58%, with humans 29% and with canids or other species 20%. The majority of participants, 80% (n = 65), saw horses for performance management either daily or weekly, while 77% (n = 62) saw horses for rehabilitation either daily or weekly (see Figure 1).

**Figure 2:**
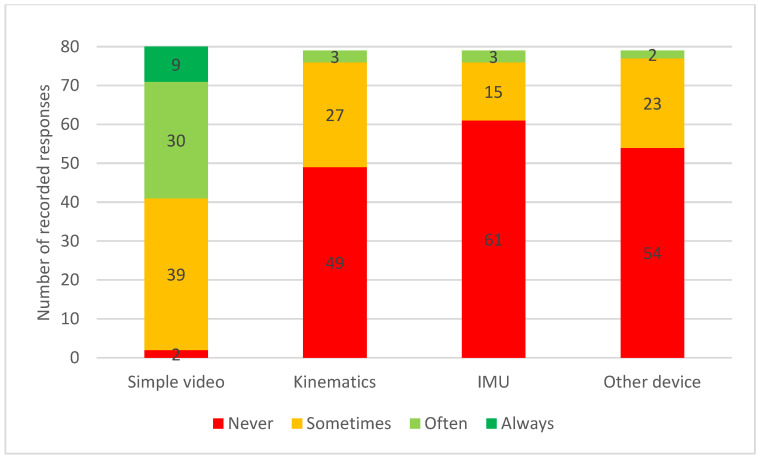
Frequency of participating equine clinicians using simple video (n = 80), kinematics (n = 79), Inertia Measurement Units (IMU) (n = 79) and other devices attached to the horse (n = 79) during assessment of quality of movement.

Simple video recordings were “always” or “often” used by 49% (n = 39) of participants while assessing quality of movement. Use of other technology was less frequent, with more than half “never” using kinematics, IMU’s or other unnamed devices (see Figure 2).

### 3.3. Essential Movement Tests

Movements on a firm surface were the most frequently used in-hand movement tests, with the use of inclines the least frequent. Movements are grouped by surface type (firm, soft or other) and displayed in the order used in the questionnaire; the cell containing the mode is highlighted/marked up (see Table 1).

Other movements suggested by participants fell into broad categories with some overlap: manual tests (n = 16 comments), particularly flexion tests (n = 4) and stimulating reflex movements (n = 5); balance (n = 13); neurological assessments (n = 12); lateral work (n = 5); under saddle (n = 2) and other (n = 10).

Rationales provided for the frequency of use of particular movements could be divided into factors related to the clinician (n = 16), the facilities (n = 13) or the horse (n = 12). Clinician factors describe un/familiarity with and preference for particular movements, as well as time limitations. Barriers to using movement tests included the facilities available, such as access to a level soft surface, inclines and poles, and horse factors such as the training level of the horse, age and discipline. Horse factors also covered the capability of the handler (n = 4) and that the clinicians’ choice of movements to observe is needs-based (n = 10). For example, the differences between a young racehorse on a yard with only access to straight concrete and an older jumping horse at an equestrian centre.

### 3.4. Patient-Specific Measures

When asked if they use formal goal setting with clients, 64% (n = 47) of participants replied in the affirmative, and 36% (n = 26) replied no. In the open-ended question, the themes raised related to the type of goal (n = 21) (being clinical/problem list, task, short/long term, performance/competition), who was involved (n = 24) (client/owner/rider, trainer/coach and veterinarian), the method—SMART (n = 8), and use of realist timeframes or feasibility of the plan (n = 17). Five participants mentioned objective measurements (time, scoring tasks, IMU data, imaging), three raised the lack of objective measures and seven described informal subjective measurement of goals.

Owner-reported measures were indicated to be used by 74% (n = 54) of participants. When asked to specify, 7 participants provided outcome measures (three- or four-point grading systems), 18 reported quantitative measures (time/sets and repetitions, heart rate, flexi-curve, competition results) and the majority (n = 44) mentioned qualitative descriptions. Themes within the qualitative descriptions included subjective descriptions of movement quality (n = 29), observation of behaviour change (n = 18), lameness (n = 4) and third-party feedback (instructor, jockey/trainer/driver, dressage score or competition results).

The monitoring of complex functional movements was reported to be via clinician visual assessment (n = 27) including repeat video assessment (n = 23) and slow-motion video (n = 4), or owner subjective report (n = 19), with a small number mentioning measurements (n = 10) such as range of motion or workload and five reporting instrumented assessment techniques (IMU, Pain trace, FES).

**Figure 3:**
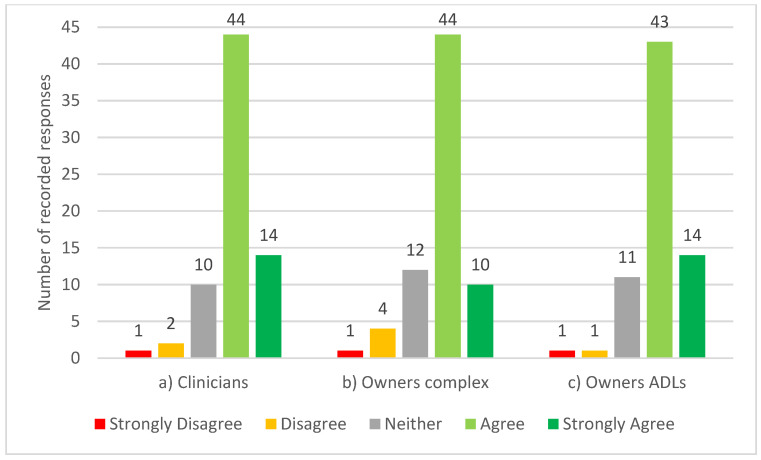
Participating equine clinicians’ opinion on the statements; “a modified equine version of the Patient-Specific Functional Scale would be useful for: (**a**) clinicians to observe complex equestrian tasks (n = 71), (**b**) owners to report complex equestrian tasks (n = 71) and (**c**) owners to report activities of daily living (ADLs)” (n = 70).

In spite of 62% (n = 45) of participants reporting familiarity with the PSFS, only 21% (n = 15) confirmed using it. A further 36% (n = 26) of the participants were unfamiliar with the PSFS. Most of the participants, 82% (n = 58), agreed, of which 20% (n = 14) strongly agreed, that a modified equine version of the PSFS for use by clinicians to observe complex equestrian tasks would be useful. A similar trend was seen for usefulness of a modified equine version of the PSFS by owners/riders to observe both complex equestrian tasks and activities of daily living (ADLs) (see Figure 3).

Comments were mostly positive, with some neutral, while some raised limitations of using owners. Positive comments focused on how the PSFS can be individualised (n = 6). Key concerns around owners using the PSFS were the difficulty of grading movement (n = 10), owners being biased (n = 4) and the lack of compliance (n = 8).

General comments in the final open-ended question were overall positive, describing the study as useful work and expressing a desire for clinically relevant outcome measures. Concerns were raised about the scope of the challenge due to the sheer volume of variables that influence movement, and suggestions were made that the focus should be on developing outcome measures for clinicians rather than owners.

### 3.5. Relationships between Variables Based on Background

Dividing participants based on undergraduate background resulted in 58% (n = 47) having a human physiotherapy background, 28% (n = 23) having a veterinary background and 15% (n = 11) having a mixed or other background.

There was a significant difference in years of experience between those with a veterinary background (19.21 years, SD = 9.7) and those with a human physiotherapy background (11.57 years, SD = 8.5)—*p* = 0.003.

The proportion of work with humans differed. Naturally, participants with a human physiotherapy background reported a larger percentage of work with humans (38%; SD = 31.82) than those from a mixed background (35%; SD = 36.83) or veterinary background (<1%; SD = 0.65) (*p* < 0.001). Those with a veterinary background attributed more of their workload to equines (88%; SD = 23.88), which significantly differed (*p* < 0.001) from the equine workload of those with a human physiotherapy background (45%; SD = 29.99) and those with a mixed background (55%; SD = 37.78).

Most participants saw rehabilitation and performance management cases regularly regardless of background discipline, be it rehabilitation (χ^2^(10, N = 81) = 8.28, *p* = 0.602) or performance management (χ^2^(10, N = 81) = 4.122, *p* = 0.942). There was no significant relationship between background and frequency of using simple video, kinematics or other devices; however, there was a statistically significant relationship (*p* = 0.009) between groups in relation to use of IMU sensors. Those with a veterinary background reported using IMU sensors “sometimes”, which was more frequently than expected, as 11/22 (50%) of the veterinary background group “sometimes” or “often” used IMUs, while only 5/46 (11%) of the human physiotherapy background group “sometimes” or “often” used IMUs. In total, 2/11 (18%) of the mixed group “sometimes” or “often” used IMUs (χ^2^(4, n = 79) = 13.485, *p* = 0.009).

In relation to the frequency of use of different movements by background profession, there were several associations between variables; however, post hoc pair-wise comparisons revealed no pattern across “always” to “never” categories.

Veterinarians were more likely to be unfamiliar with the PSFS than allied health professionals, with a significant difference between groups (*p* ≤ 0.001). Those with a physiotherapy background are both more likely to use the PSFS (14/39; z = −2.72, *p* ≤ 0.001) and less likely to be unfamiliar with the PSFS (4/39; z = 2.607, *p* ≤ 0.001). Those with a veterinary background are both more likely to be unfamiliar with the PSFS (18/22; z = 3.503, *p* ≤ 0.001) and less likely to be familiar with but not use the PSFS than statistically expected (3/22; z = −2.065, *p* ≤ 0.001).

There was no significant relationship between background and opinion on the usefulness of the PSFS by clinicians or owners. Those with fewer years of experience were more likely to be familiar with or use the PSFS (*p* = 0.043). All others relationships tested for, including for themes in the open-ended responses, found no significant difference.

## 4. Discussion

This survey is the first time equine clinicians have been asked about assessing quality of movement and, in contrast to existing lameness studies, this survey was multidisciplinary, consulting veterinarians and allied health professionals. In human medicine, siloed healthcare is being discouraged [39], and multidisciplinary practice is the future. Grading lameness is different to additionally assessing quality of movement; therefore, this list of key movements is a springboard to developing a unique outcome measure. There is little published research regarding quality of movement, including more complex movement, although Hobbs’s scoping study [45] discusses it in relation to performance. Despite the professions having differing aims, these results demonstrate similarities between veterinarians and physiotherapists in the frequency of use of movements, which raises hopes regarding greater teamwork and complementary practice.

When the literature revealed no standardized set of movement tests for evaluating lameness or quality of movement, consulting a focus group of experts was considered, but it was decided a survey would canvas a larger audience and likely be more representative of field-based practice.

### 4.1. Participant Characteristics

The majority of participants saw equine performance management and rehabilitation cases either daily or weekly, thus regularly engaging with the type of horses this new outcome measure is intended for. In this sample, there was no difference between veterinarians and allied health professionals in seeing rehabilitation cases; however, that may be attributed to this survey targeting equine sports medicine and rehabilitation diplomats, not general veterinarians.

Simple video recordings are easily accessible via smart phones, compared to more expensive IMU systems, some of which are only available to veterinarians. It appears that many clinicians do not have access to instrumented gait analysis despite new systems becoming more readily available [46]. While the use of additional apps or software, e.g., to measure angles in videos, was not ascertained, the widespread use of video is a positive sign. Devices were reported to be used “sometimes” or “often”, implying that they are not advantageous for assessing quality of movement with all equine clients. There was no significant relationship between those using devices and responses to other questions, such as preference for modifying the PSFS, suggesting access to technology did not bias participants.

### 4.2. Movement Test Preference

A list of the key movements most commonly used when assessing quality of movement has been collated from practicing equine clinicians. While previous studies had stated trot in a straight line was insufficient [23,47], a broader range of movements had not been defined. The movements listed are conducted in-hand, which helps bridge the gap between passive movements, or manually applied pressures, and a ridden assessment. These results support lists of common movements used for grading lameness [28], but refines those lists into movements used “always” or “often” to assess quality of movement.

Information on how complex movement is currently measured has been gathered, and although there were few objective outcome measures used for assessing complex movement, clinicians are keen for these to be developed further. Equine clinicians reported wanting to be more objective, and acknowledged the subjectivity and limitations of current assessment practices. This sentiment is in line with responses from veterinary physiotherapists in the United Kingdom [32], which highlighted the lack of objective measures, the level of understanding of the differences between subjective and objective measures, and the desire for more outcome measures to be designed for clinical practice, not just research.

The most commonly used movements and the barriers to assessment, while predictable, are now formally supported by survey evidence upgrading previously anecdotal assertions. A repeated theme in the comments was that assessment is an iterative process, with movements being chosen on a case-by-case basis. Despite this, within the frequently observed movements there appears to be a key group routinely used by equine clinicians. Several movements on a firm surface scored a mode of “always”; these are walk and trot in a straight line and on a small circle, step back and hind leg cross-over. In the context of fully assessing quality of movement and designing a suitable outcome measure, this seems a limited set of movements. In addition to the above “always”-observed movements, the larger subgroup that received a classification of “often” branches out to include different surfaces and gaits.

Movements scoring a mode of “never” or “sometimes” fit with the barriers mentioned previously, as these movement tasks require equipment (e.g., poles) or facilities (e.g., inclines) and a greater skill level from the horse and handler (e.g., front leg cross-over). Some clinicians described their assessment as restricted by industry expectations; for example, limited time per horse in racing stables, with the expectation to abbreviate assessment to palpation of the horse in the stable and not observing functional movements, in comparison to thoroughly assessing a dressage/sports horse all the way through being tacked up and ridden through specific movements reported by the rider as suboptimal.

Confining a new outcome measure to active in-hand movement tests avoids additional tack and rider related factors, or tests involving manual pressure (such as provoking balance reactions or stimulating muscular reflexes), which are difficult to standardise. Active in-hand movements are directed or guided by a cue, not by manual pressure. In addition, tools such as range of motion goniometers and palpation scales [37] already exist for hands-on assessment, along with the Ridden Horse Pain Ethogram [48], for assessing ridden behaviours. While acknowledging that pain, asymmetry, lameness, behaviour, and performance are inter-related constructs, this new outcome measure will focus on quality of movement or movement dysfunction. Dynamic mobilisation exercises were included in the survey and had a mode of “often”; however, while they are active in-hand movements, they differ from the other movement tests, as they are non-ambulatory and therefore should be excluded from further development within a new quality of movement outcome measure.

When asked to suggest other movement tests (or to identify any missing), participants’ responses fell into categories of neurological tests (head high walking, tail pull), manual tests (weight shifting, resistance to displacement, hop test), reflexes (myotactic rounding response), manipulative assessments (flexion tests), three-leg balance, lateral work in-hand (which requires more advanced training in horse and handler to provide accurate pressure and release cues), under saddle active movements, and passive movements such as limb range of movement and back wiggle using the tail head. Participants also suggested observing movements the horse owner reported they are having issues with, such as tacking up, which are more behavioural assessments rather than quality of movement-related, but they are certainly a part of the observation phase of assessment and have been investigated by others, e.g., [49]. Many of the stimulated responses, passive, assisted or facilitated movements suggested can be influenced by the horse’s motivation level and the applied external pressure, and are therefore difficult to standardise. Flexion tests have known issues with standardisation of force, time and individual horse response [50,51,52,53]; although they are not an accurate diagnostic tool [54], they are nonetheless still used to indicate areas of interest. However, it should be noted that the new outcome measure being designed is not intended for diagnosis, and flexion tests will not be included in it. Observing specific owner-reported movements comes under the remit of a modified PSFS, while balance and neurological tests are suitable for future research to create an equine neurological battery, similar to the canine FINFUN [55]. Reliable methods of monitoring canine functional movement are more advanced, with several condition specific outcome measures already in use e.g., neurological, arthritis, stifle and chronic pain [56].

Participants did not mention lunging (15–20 m circles) or cantering on the firm surface, only reporting small circles (5–10 m) at walk and trot. Due to its speed and biomechanics, canter places higher forces through the limbs, and cantering on a firm surface may pose an increased risk of exacerbating an issue and risk of slipping due to decreased traction on a smooth surface. Observing canter is useful, particularly for back pain and hindlimb lameness [57], but safety must always be considered first.

### 4.3. Patient-Specific Measure Preferences

The majority of therapists stated that they use formal goal-setting, sometimes with separate goals for owners and the clinician. However, without measurement, they are not outcome measures and lack the strong reliability, validity and sensitivity of systems such as goal attainment scaling (GAS) [58]. GAS helps with setting realistic goals [59]; being realistic was repeatedly mentioned in the survey responses. While SMART goals are supposed to be measurable, the achievement of many goals is often all or nothing [58]. No participants mentioned the use of GAS, nor any way of applying a weighting to reflect the importance or difficulty of the goal. However, GAS is time-consuming [60], which is one of the main reasons equine physiotherapists mention for not using objective measures [32].

The PSFS is a streamlined version of monitoring goals, with the 0–10 scoring being simpler for clients, but still incorporating discussion of what is realistic, and by setting what a 10/10 performance would look like, the individual’s current ability and the steps necessary to bridge the gap are made clear. The PSFS is used in human physiotherapy, so it is unsurprising that fewer veterinarians had heard of the PSFS, and if a clinician is unfamiliar with an outcome measure, they are less likely to use it [61]. If all those involved in equine care embrace the new outcome measure, their ability to communicate with each other will be enhanced.

Equine clinicians are predominately using subjective methods to monitor complex movements, such as observation (live or video) and reported competition performance. Several participants stated they used objective measurements where possible, but admitted they were currently lacking. Many acknowledged that their approach taken to goal-setting and monitoring complex movements was informal, heavily reliant on subjective reports from the owner or the clinician’s opinion, and not objectively measured or scored. When dealing with owners, a couple of participants mentioned using variations of a Likert grading scale (same/better/worse/different). Generally, these are simple to use as they indicate direction but not magnitude. The majority of participants supported the idea of modifying the PSFS for use with horses. Comments revealed that owners are not trusted to assess movement quality, yet for client-centred care, more education and involvement of owners is desirable [39].

### 4.4. Limitations and Further Research

The number of participants exceeded the a priori calculation of what was required to give the study acceptable power; nevertheless, sample size and self-selection bias must be acknowledged as potential limitations regarding the generalisability of the research findings reported in this study. The number of valid responses, from participants with comparable qualifications and experience in rehabilitation and performance management, would suggest that outcomes are associated with external validity. The number of participants attracted was similar to that in Tabor and Williams’ [32] survey of veterinary physiotherapists in the United Kingdom, which used snowball sampling. Interest in the topic and survey fatigue likely contribute to the small participant numbers. Overall, the reach of the survey was limited by relying on third parties (professional associations) to distribute the link, and those parties not being personally invested or benefitting from ensuring distribution to all their members. Furthermore, some countries do not appear to have national associations, some members email address maybe incorrect, and some associations’ rules around frequency of contacting members may mean potential participants only saw the link once, perhaps within a scheduled monthly e-newsletter. The high completion and completeness rates (94% and 90%) indicate strong engagement from those who chose to participate. The high average years of practice (13.9 years) and multiple qualifications support the participants having valuable clinical experience and knowledge that will inform their responses. Predictably, many of the participants also worked with humans, as in some countries this is a requirement to maintain their human physiotherapy registration. Even with restricting the inclusion criteria, there were over 30 different qualifications reported within this sample, with participants coming form 14 different countries. While the results of this study should be viewed through a descriptive lens, the coherence of the responses suggests similar assessment practices and challenges across the globe. Further research could look to replicate the findings with a broader population of equine physiotherapists and equine veterinarians.

This summary of the opinions of 81 equine clinicians advances previous knowledge by identifying a group of key movements to observe that can be taken forward as the foundation for a new quality of movement outcome measure. Despite the small number of objective measures in use, there is a strong desire for more robust outcome measures that can be integrated into practice. Future research efforts should focus on adapting the PSFS, currently in use in human medicine [62], for use with horses. Although research published on lameness has progressed substantially with instrumented techniques, there is still much that can be done to improve field-based visual observations of simple and complex movements.

## 5. Conclusions

An online survey of equine clinicians identified the most frequently observed in-hand movements, with a key group of 24 observed “always” or “often”. The main limiting factors reported for assessments were the availability of different surfaces, and the horse and handler training level. Participants perceive benefits in modifying the Patient-Specific Functional Scale for monitoring complex movements. These movements will be taken forward for refinement as a battery of field-based quality of movement tests, accompanied by a modified PSFS for specific individual goals. Equine clinicians are keen for new outcome measures to be developed, but concerned that they need to be not only valid and reliable, but also user-friendly. Creating a new quality of movement outcome measure for horses undergoing performance management or rehabilitation will improve the ability to assess treatment efficacy, therefore enhancing evidence-based practice.

## Figures and Tables

**Table 1:** Frequency of use of in-hand movement tests when assessing equine quality of movement. Mode indicated with * and shaded (green for a mode of always, yellow for a mode of often, orange for a mode of sometimes and red for a mode of never), percentages < 50% shaded grey (n = 73).

	Frequency of Use				
Movement Test	Never	Sometimes	Often	Always	Percentages of Often and Always Combined (%)
**Firm Surface**					
Walk in a straight line—viewed from behind		4	8	61 *	95
Walk in a straight line—viewed from in front	1	6	7	59 *	90
Walk in a straight line—viewed side-on		9	15	49 *	88
Trot in a straight line—viewed from behind		8	17	48 *	89
Trot in a straight line—viewed from in front		10	17	46 *	86
Trot in a straight line—viewed side-on		14	27	32 *	80
Small circle (5–10 m) at the walk left and right rein	3	18	22	30 *	71
Small circle (5–10 m) at the trot left and right rein	4	23 *	23 *	23 *	63
Rein back/step back		20	14	39 *	73
Pivot/turn on the forehand left and right (aka hind leg cross-over, yielding the hind quarters)	5	21	15	32 *	64
Front leg cross-over/yielding the shoulders	11	28 *	15	19	47
Figure of 8/change of bend using tight turns (<5 m) in walk	17	31 *	19	6	34
**Soft Surface**					
Walk in a straight line—viewed from behind	4	15	38 *	16	74
Walk in a straight line—viewed from in front	4	16	38 *	15	73
Walk in a straight line—viewed side-on	5	18	37 *	13	68
Trot in a straight line—viewed from behind	3	16	41 *	13	74
Trot in a straight line—viewed from in front	3	20	38 *	12	68
Trot in a straight line—viewed side-on	3	22	39 *	9	66
Small circle (5–10 m) at the walk left and right rein	5	26	33 *	9	58
Small circle (5–10 m) at the trot left and right rein	7	23	35 *	8	59
Rein back/step back	8	26 *	26 *	13	53
Lunged on a circle (~15–20 m) at walk left and right rein	6	23	26 *	18	60
Lunged on a circle (~15–20 m) at trot left and right rein	7	21	26 *	19	62
Lunged on a circle (~15–20 m) at canter left and right rein	7	23	26 *	17	59
**Other**					
Walk up and down an incline	5	52 *	14	2	22
Lunge on an incline	36 *	33	3	1	5
Walk over pole/s	13	46 *	13	1	19
Trot over pole/s	16	47 *	9	1	14
Transition from halt to walk	7	21	23 *	22	62
Transition from walk to trot	4	24 *	24 *	21	62
Transition from trot to canter left/right lead on the lunge	4	30 *	26	13	53
Transition from canter to trot	3	32 *	26	12	52
Transition from trot to walk	3	25 *	22	23	62
Transition from walk to halt	6	24 *	21	22	59
Transition walk to halt on a diagonal line down incline	51 *	19	2	1	4
Transition halt to walk on a diagonal line up an incline	51 *	18	2	2	5
Dynamic mobilisations/baited stretches flexion/extension plane	2	14	31 *	26	78
Dynamic mobilisations/baited stretches lateral flexion and rotation left and right	1	15	30 *	27	78

**Figure A1:**
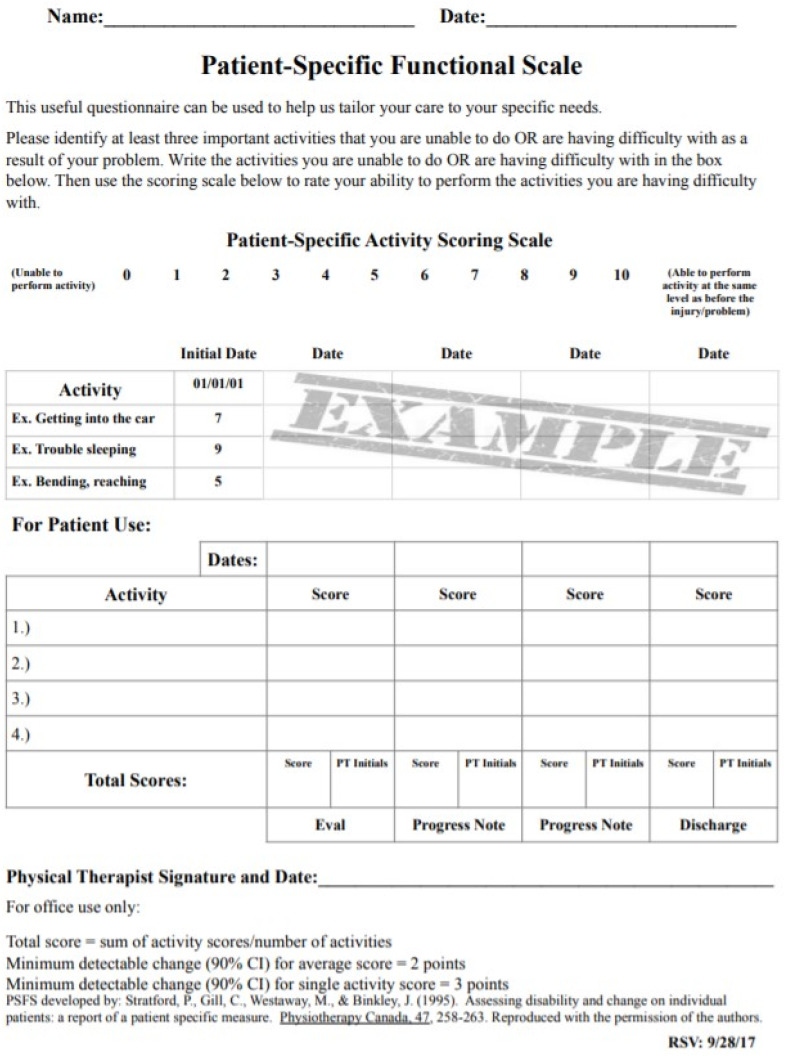
The Patient-Specific Functional Scale.

## Data Availability

Deidentified data are only available on request via Charles Sturt University, through Hayley Randle.

## Supplementary Materials

The following supporting information can be downloaded at: https://www.mdpi.com/article/10.3390/ani13182822/s1, Questionnaire—Functional movement outcome measures in equine physiotherapy and rehabilitation.
