# Supplementary material for: Visually Assessing Equine Quality of Movement: A Survey to Identify Key Movements and Patient-Specific Measures
Source: Animals (Basel). 2023 Sep 5;13(18):2822. doi: 10.3390/ani13182822 (PMC10525514; doi:10.3390/ani13182822)

Charles Sturt University

Functional Movement Outcome Measures in Equine Physiotherapy and Rehabilitation

Participant Information Statement

Invitation

You are invited to participate in a study that aims to develop an outcome measure to assess horses' quality of movement.

This research is being conducted by Annette Bowen, PhD student, and an international multidisciplinary team including Physiotherapists, a Veterinarian and an Equine Scientist:

Annette Bowen, PhD student
School of Agricultural, Environmental and Veterinary Sciences
Charles Sturt University
Email: abowen@csu.edu.au

Assoc. Professor Hayley Randle
School of Agricultural, Environmental and Veterinary Sciences
Charles Sturt University
Email: hrandle@csu.edu.au

Assoc. Professor Raphael Labens
School of Agriculture, Environmental and Veterinary Sciences
Charles Sturt University
Email: rlabens@csu.edu.au

Dr Gillian Tabor
Senior Lecturer, Equine Rehabilitation and Physiotherapy
Hartpury University
Gloucester
United Kingdom
Email: gillian.tabor@hartpury.ac.uk

Before you decide whether or not you wish to participate in this study, it is important for you to understand why the research is being done and what it will involve. Please take the time to read the following information carefully and discuss it with others if you wish.

What is the purpose of this study?
This survey is part 2 of a project aiming to develop an outcome measure to assess horses' quality of movement (see figure 1). The purpose of the survey is to determine what functional movement tests equine clinicians think are most useful to include in the outcome measure. Your opinion will also be sought on modifying an existing human physiotherapy outcome measure for use with horses.

Your input is vital to this study.

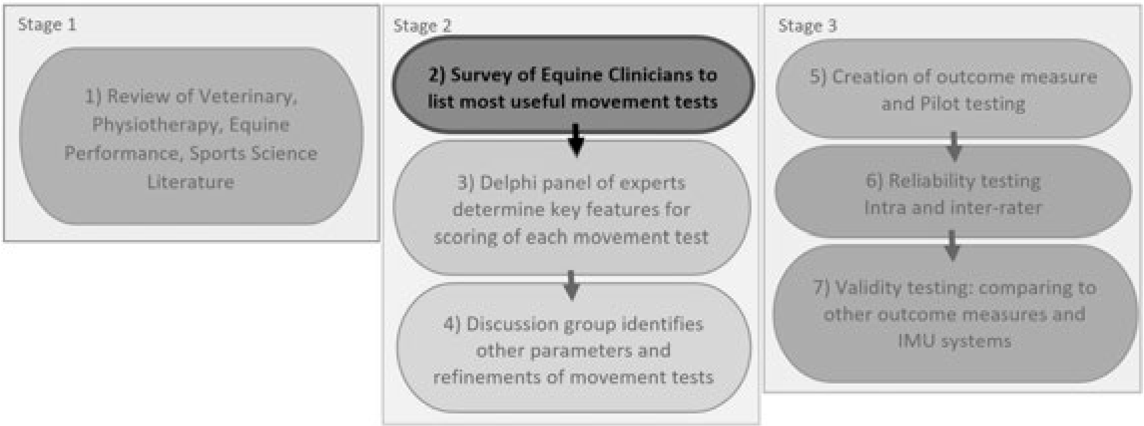

Figure 1. Flowchart of research steps.

The proposed outcome measure is a battery of movement tests for use in the field, to monitor movement status in response to interventions. Rather than a global score, as in a traditional lameness trot-up, each movement test will be scored individually to more precisely convey where gains or losses are occurring. The outcome measure may be used independently, in conjunction with, or to complement screening with objective quantitative technology.

The outcome measure is for use by equine clinicians working with horses undergoing rehabilitation or managing performance issues. Such horses may present with movement dysfunction, motion asymmetry, inconsistent, subtle or mild lameness (e.g. AAEP <2 Lameness that is difficult to observe at a walk or trot in a straight line but is present under certain circumstances). The battery will be confined to active in-hand movement tests, to avoid additional tack and rider related factors, and tests involving manual pressure (such as provoking balance reactions or stimulating muscular reflexes) are more difficult to standardise. Whilst acknowledging that pain, behaviour, asymmetry, lameness and performance are inter-related constructs, this outcome measure will focus on movement, and in particular quality of movement or movement dysfunction.

Why have I been invited to participate in this study?
We are seeking equine clinicians experienced in the areas of rehabilitation and performance. Professional associations of Equine Sports Medicine and Rehabilitation Veterinarians, Animal/Veterinary/Equine Physiotherapists and Animal Biomechanical Professionals (Chiropractors, Osteopaths) have therefore been approached to distribute the survey link to their members.

If you do not have at least an undergraduate degree in a relevant field, you are unfortunately not eligible to participate. This requirement is to try to retain similarity in clinical reasoning and assessment processes.

What does this study involve?
If you agree to participate you will be asked to give your consent at the beginning of the short questionnaire. The questionnaire has questions divided into 3 parts: 1st - demographics to record your training and experience, 2nd - rating the usefulness of movement tests and 3rd - your opinion of an already existing functional scale.

It is estimated the questions will take 10-15 minutes to answer. The survey will be open until Monday 4th July.

Are there risks and benefits to me in taking part in this study?
There are no foreseen risks to participation. We cannot promise you any direct benefits from participating in this survey. You may opt-in to receive a summary of the results and links to any future papers published by emailing abowen@csu.edu.au. An outcome measure to monitor equine movement dysfunction will support equine welfare, by assisting clinicians improve treatment efficacy in the field and build evidence for treatment techniques. Your assistance now will help shape the outcome measure to be relevant to your clinical needs by informing which core movement tests to include.

How is this study being paid for?
This project is supported by an Australian Government Research and Training Scholarship. This organisation will have no input to the research results.

Will taking part in this study cost me anything, and will I be paid?
There are no costs or payments for participating in this survey and your participation is voluntary. You may choose to take part as the social exchange of research.

What if I don’t want to take part in this study?
Participation in this research is entirely your choice. Only those people who give their informed consent will be included. Whether or not you decide to participate is your decision and not doing so will not disadvantage you in any way.

What if I participate and want to withdraw later?
You may choose to stop participating at any point. Responses are recorded as you move through the questions. However, as data are collected anonymously, once you have submitted an answer your responses cannot be withdrawn.

How will confidentiality be protected?
The survey is anonymous and it will not be possible to identify you from your answers. Data will be retained for up to 5 years and held securely on the secured servers at Charles Sturt University. Only the lead researcher and primary supervisor will have access to the data. The data will also be stored within the SurveyMonkey© platform according to their Privacy Policy and Security Statement. The list of email addresses to send summary results to will be stored in a separate password protected file and not shared or sold on to any third party. After a 5 year period the file of email addresses will be deleted.

What will happen to the information that I give you?
The data collected will be analysed and the findings used to inform development of the proposed outcome measure. Summary demographic data about profession and experience will be reported. Individuals will not be identified. A summary of results will be returned to participants who request it. The results will be submitted as part of Annette Bowen's PhD thesis. Further to this, the research findings may be presented at conferences or published as articles in peer-reviewed journals.

What should I do if I want to discuss this study further before I decide?
If you would like further information, please contact Annette Bowen via email: abowen@csu.edu.au

Who should I contact if I have concerns about the conduct of this study?
Charles Sturt University's Human Research Ethics Committee has approved this project [Protocol Number: H22082]. If you have any complaints or reservations about the ethical conduct of this project, you may contact the Committee through the Ethics and Compliance Unit via the following contact details:

The Presiding Officer
Human Research Ethics Committee
Ethics and Compliance Unit
Locked Bag 588
Wagga Wagga NSW 2678
Phone: (02) 6933 4213
Email: ethics@csu.edu.au

Any issues you raise will be treated in confidence and investigated fully and you will be informed of the outcome.

Please print this information sheet if you wish to keep a copy for your records (use the Print option from the browser File menu).

Charles Sturt University

Functional Movement Outcome Measures in Equine Physiotherapy and Rehabilitation

Consent

Please ensure you have read the previous information about this survey before giving your consent or answering the questions.

- I confirm I am aged over 18 years.
- I agree to participate in the research project described and give my consent freely.
- I have read and understand the Participant Information provided to me, which I have had the opportunity to retain (save or print).
- The purpose of the research, the procedures required, and the possible risks and benefits have been explained to me.
- I have been given the opportunity to ask questions about the research and received satisfactory answers.
- By proceeding with this survey, I give my consent for my responses to be stored and used by the research team as described above.
- I understand once responses are submitted they are unable to be withdrawn.
- I consent to participating in this survey.

If you are happy to proceed to the survey, please click the '**Next**' button below.

Charles Sturt University

Functional Movement Outcome Measures in Equine Physiotherapy and Rehabilitation

Section 1: Demographic Information

1. What undergraduate qualifications do you have?
(Please select all that apply)

- [ ] Veterinary Medicine
- [ ] Human Physiotherapy
- [ ] Human Chiropractic
- [ ] Human Osteopathy
- [ ] Veterinary Physiotherapy
- [ ] Other (please specify)

2. What postgraduate training do you have as an equine clinician?
(Please select all that apply)

- [ ] Veterinary Sports Medicine and Rehabilitation Diplomate
- [ ] Masters - Veterinary / Animal / Equine Physiotherapy
- [ ] Post Graduate Diploma - Veterinary / Animal / Equine Physiotherapy
- [ ] Post Graduate Diploma - Animal Biomechanical Professional
- [ ] Masters / Post Graduate Diploma - Veterinary / Animal / Equine Chiropractics
- [ ] Certificate / Diploma - Veterinary / Animal / Equine Osteopathy
- [ ] Certificate / Diploma - Equine Physiotherapy / Rehabilitation
- [ ] Other (please specify)
- [ ] None

3. How many years have you been practising as an equine clinician?
(Please enter years as a whole number)

4. In what country are you mainly based?

Charles Sturt University

Functional Movement Outcome Measures in Equine Physiotherapy and Rehabilitation

5. Approximately what proportion of your work is with humans, equines, and canines/other animals?

% Humans:

% Equine:

% Canine / other:

Rehabilitation refers to the recovery from injury or disease.
Performance relates to managing the stresses and compensations associated with intense physical exercise and high-level athletic performance.

6. Within your equine work, how often would you see cases for rehabilitation or performance management?

	Daily	Weekly	Monthly	Quarterly	Annually	Never
Rehabilitation:	○	○	○	○	○	○
Performance management:	○	○	○	○	○	○

7. In your equine practice do you use any of these technologies to assist you in assessing movement quality, and if so, how often?

	Never	Sometimes	Often	Always
Simple video (e.g. smart phone or camera)	○	○	○	○
Kinematic analysis, markers on the horse	○	○	○	○
IMU sensors on the horse	○	○	○	○
Other device on the horse, e.g. on the saddle or girth, horseshoes	○	○	○	○

Charles Sturt University

Functional Movement Outcome Measures in Equine Physiotherapy and Rehabilitation

Section 2: Movement Tests

These questions are to determine the core movement tests you believe are most useful when assessing quality of movement or movement dysfunction. Remembering the new outcome measure is not intended to be a lameness work up, but to monitor any change in movement status pre and post intervention. Lameness, asymmetry and behavioural indicators of discomfort may be used as signs of movement dysfunction and scoring criteria will be developed in subsequent stages of the project.

Note: We suggest briefly skimming the list before beginning to enter your responses. An opportunity to add other tests will follow.

* 8. Please indicate how frequently you use each movement test listed below:

ON A FIRM SURFACE

	Never	Sometimes	Often	Always
Walk in a straight line - viewed from behind	○	○	○	○
Walk in a straight line - viewed from in front	○	○	○	○
Walk in a straight line - viewed side on	○	○	○	○
Trot in a straight line - viewed from behind	○	○	○	○
Trot in a straight line - viewed from in front	○	○	○	○
Trot in a straight line - viewed side on	○	○	○	○
Small circle (5-10m) at the walk left and right rein	○	○	○	○
Small circle (5-10m) at the trot left and right rein	○	○	○	○
Rein back / step back	○	○	○	○
Pivot/ turn on forehand left & right (aka hind leg cross over, yielding the hind quarters)	○	○	○	○
Front leg cross over/ yielding the shoulders	○	○	○	○
Figure 8 / change of bend using tight turns (<5m) in walk	○	○	○	○

* 9. Please indicate how frequently you use each movement test listed below:

ON A SOFT SURFACE

	Never	Sometimes	Often	Always
Walk in a straight line - viewed from behind	○	○	○	○
Walk in a straight line - viewed from in front	○	○	○	○
Walk in a straight line - viewed side on	○	○	○	○
Trot in a straight line - viewed from behind	○	○	○	○
Trot in a straight line - viewed from in front	○	○	○	○
Trot in a straight line - viewed side on	○	○	○	○
Small circle (5-10m) at the walk left and right rein	○	○	○	○
Small circle (5-10m) at the trot left and right rein	○	○	○	○
Rein back / step back	○	○	○	○
Lunged on a circle (~15-20m) at walk left and right rein	○	○	○	○
Lunged on a circle (~15-20m) at trot left and right rein	○	○	○	○
Lunged on a circle (~15-20m) at canter left and right rein	○	○	○	○

* 10. Please indicate how frequently you use each movement test listed below:

	Never	Sometimes	Often	Always
Walk up and down an incline	◯	◯	◯	◯
Lunge on an incline	◯	◯	◯	◯
Walk over pole/s	◯	◯	◯	◯
Trot over pole/s	◯	◯	◯	◯
Transition from halt to walk	◯	◯	◯	◯
Transition from walk to trot	◯	◯	◯	◯
Transition from trot to canter left/right lead on the lunge	◯	◯	◯	◯
Transition from canter to trot	◯	◯	◯	◯
Transition from trot to walk	◯	◯	◯	◯
Transition from walk to halt	◯	◯	◯	◯
Transition from walk to halt on a diagonal line down an incline	◯	◯	◯	◯
Transition from halt to walk on a diagonal line up an incline	◯	◯	◯	◯
Dynamic mobilisations / baited stretches flexion/extension plane	◯	◯	◯	◯
Dynamic mobilisations / baited stretches lateral flexion & rotation left and right	◯	◯	◯	◯

11. Please add any other in-hand tests you use:

12. Please provide any comments or rationale for your answers above, including if you are not familiar with any of the movement tests:

Charles Sturt University

Functional Movement Outcome Measures in Equine Physiotherapy and Rehabilitation

Section 3: Measuring Goals

As stated previously, the scope of the new outcome measure will be confined to a core group of active in-hand movement tests, excluding ridden assessment and the use of manual pressure. During the process of rehabilitation or optimising performance, more complex equestrian tasks specific to the individual also need to be evaluated.

The literature suggests there is minimal use of outcome measures in equine physiotherapy and rehabilitation to assess functional movements, activity limitations and participation restrictions. This section seeks to understand how some of these complex movements may be assessed.

13. Formal goal setting incorporates shared decision making and monitoring of progress or attainment of goals.
For complex equestrian tasks specific to the individual client, do you use formal goal setting?

- ◯ Yes
- ◯ No

14. Please specify the approach taken to goal setting:

Charles Sturt University

Functional Movement Outcome Measures in Equine Physiotherapy and Rehabilitation

15. Do you use any owner reported measures?

- ◯ Yes
- ◯ No

Functional Movement Outcome Measures in Equine Physiotherapy and Rehabilitation

16. Please specify the owner reported measures you use:

Charles Sturt University

Functional Movement Outcome Measures in Equine Physiotherapy and Rehabilitation

17. What, if anything, do you use to monitor complex functional movements, activity limitations or participation restrictions?

Charles Sturt University

Functional Movement Outcome Measures in Equine Physiotherapy and Rehabilitation

Patient-Specific Functional Scale (PSFS)

Name:________________________________ **Date:**__________________________

Patient-Specific Functional Scale

This useful questionnaire can be used to help us tailor your care to your specific needs.

Please identify at least three important activities that you are unable to do OR are having difficulty with as a result of your problem. Write the activities you are unable to do OR are having difficulty with in the box below. Then use the scoring scale below to rate your ability to perform the activities you are having difficulty with.

Patient-Specific Activity Scoring Scale

(Unable to perform activity)	0	1	2	3	4	5	6	7	8	9	10	(Able to perform activity at the same level as before the injury/problem)

	Initial Date	Date	Date	Date	Date
**Activity**	01/01/01				
Ex. Getting into the car	7				
Ex. Trouble sleeping	9				
Ex. Bending, reaching	5				

EXAMPLE

For Patient Use:

	Dates:								
**Activity**		Score		Score		Score		Score	
1.)									
2.)									
3.)									
4.)									
**Total Scores:**		Score	PT Initials	Score	PT Initials	Score	PT Initials	Score	PT Initials
		Eval		Progress Note		Progress Note		Discharge	

Physical Therapist Signature and Date:____

For office use only:

Total score = sum of activity scores/number of activities
Minimum detectable change (90% CI) for average score = 2 points
Minimum detectable change (90% CI) for single activity score = 3 points
PSFS developed by: Stratford, P., Gill, C., Westaway, M., & Binkley, J. (1995). Assessing disability and change on individual patients: a report of a patient specific measure. Physiotherapy Canada, 47, 258-263. Reproduced with the permission of the authors.

RSV: 9/28/17

18. How familiar are you with the Patient-Specific Functional Scale (PSFS) as used in human physiotherapy?

- ◯ I am familiar with the PSFS and use it
- ◯ I am familiar with the PSFS but don't use it
- ◯ I am not familiar with the PSFS

Charles Sturt University

Functional Movement Outcome Measures in Equine Physiotherapy and Rehabilitation

19. Please indicate how you feel about each of the following statements:

	Stongly disagree	Disagree	Neither agree nor disagree	Agree	Strongly agree
A modified equine version of the PSFS for use by clinicians to observe complex equestrian tasks would be useful.	○	○	○	○	○
A modified equine PSFS for use by owners/riders/carers to observe complex equestrian tasks would be useful.	○	○	○	○	○
A modified equine PSFS for use by owners/riders/carers to observe activities of daily living would be useful.	○	○	○	○	○

20. Please provide any comments or rationale for your responses:

21. Is there anything else you would like to add?
Please enter further comments, suggestions or other information:

Charles Sturt University

Functional Movement Outcome Measures in Equine Physiotherapy and Rehabilitation

Survey End

You have now completed the questions.

Thank you for taking part in this survey, your responses are appreciated.

The results will help inform the development of an outcome measure for assessing equine functional movement.

If you have any further questions or comments, you can contact the lead researcher via abowen@csu.edu.au

If you would like to receive a summary of the results and a link to any future papers published, please send an email to abowen@csu.edu.au with subject “Survey Summary Request”. Your email will not be linked in any way to your survey responses which remain anonymous.

Please click the '**Done**' button below to finalise your survey.

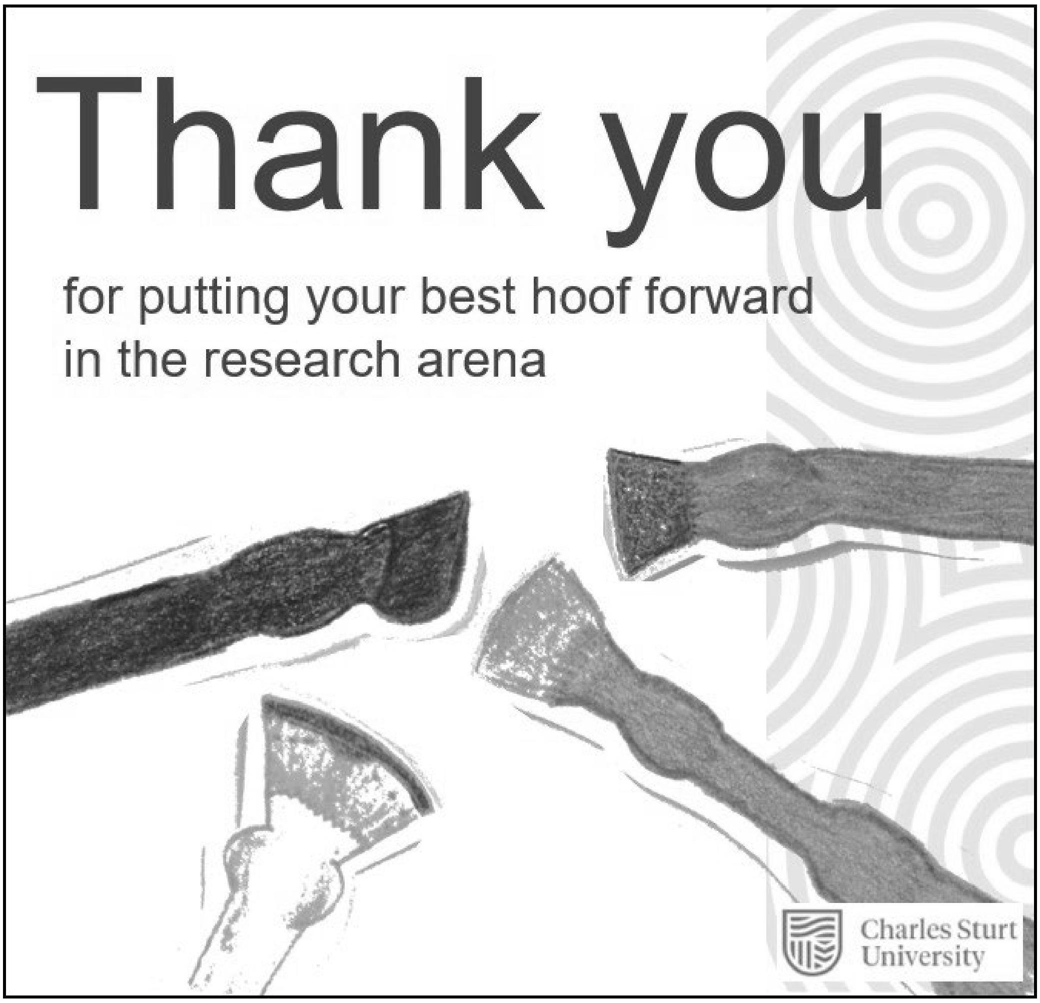

Supplement: Supplementary file 1 [file animals-13-02822-s001.zip › animals-2528729-supplementary.pdf]
